# Effect of angiotensin‐converting enzyme inhibition on cardiovascular adaptation to exercise training

**DOI:** 10.14814/phy2.15382

**Published:** 2022-07-13

**Authors:** Tórur Sjúrðarson, Jacob Bejder, Andreas Breenfeldt Andersen, Thomas Bonne, Kasper Kyhl, Tóra Róin, Poula Patursson, Noomi Oddmarsdóttir Gregersen, May‐Britt Skoradal, Michael Schliemann, Malte Lindegaard, Pál Weihe, Magni Mohr, Nikolai B. Nordsborg

**Affiliations:** ^1^ Center of Health Science, Faculty of Health Science University of the Faroe Islands Tórshavn Faroe Islands; ^2^ Department of Nutrition, Exercise, and Sports (NEXS) University of Copenhagen Copenhagen Denmark; ^3^ Department of Cardiology at Copenhagen University Hospital Rigshospitalet Copenhagen Denmark; ^4^ Department of Surgery, The Faroese Hospital System Tórshavn Faroe Islands; ^5^ FarGen, The Genetic Biobank of the Faroe Islands Tórshavn Faroe Islands; ^6^ Department of Occupational Medicine and Public Health The Faroese Hospital System Tórshavn Faroe Islands; ^7^ Department of Sports Science and Clinical Biomechanics, SDU Sport and Health Sciences Cluster (SHSC) Faculty of Health Sciences, University of Southern Denmark Odense Denmark

**Keywords:** angiotensin‐converting enzyme inhibitors, cardio‐vascular health, exercise, hypertension

## Abstract

Angiotensin‐converting enzyme (ACE) activity may be one determinant of adaptability to exercise training, but well‐controlled studies in humans without confounding conditions are lacking. Thus, the purpose of the present study was to investigate whether ACE inhibition affects cardiovascular adaptations to exercise training in healthy humans. Healthy participants of both genders (40 ± 7 years) completed a randomized, double‐blind, placebo‐controlled trial. Eight weeks of exercise training combined with placebo (PLA, *n* = 25) or ACE inhibitor (ACEi, *n* = 23) treatment was carried out. Before and after the intervention, cardiovascular characteristics were investigated. Mean arterial blood pressure was reduced (*p* < 0.001) by −5.5 [−8.4; −2.6] mmHg in ACE_i_, whereas the 0.7 [−2.0; 3.5] mmHg fluctuation in PLA was non‐significant. Maximal oxygen uptake increased (*p* < 0.001) irrespective of ACE inhibitor treatment by 13 [8; 17] % in ACE_i_ and 13 [9; 17] % in PLA. In addition, skeletal muscle endurance increased (*p* < 0.001) to a similar extent in both groups, with magnitudes of 82 [55; 113] % in ACE_i_ and 74 [48; 105] % in PLA. In contrast, left atrial volume decreased (*p* < 0.05) by −9 [−16; −2] % in ACE_i_, but increased (*p* < 0.01) by 14 [5; 23] % in PLA. Total hemoglobin mass was reduced (*p* < 0.01) by −3 [−6; −1] % in ACE_i_, while a non‐significant numeric increase of 2 [−0.4; 4] % existed in PLA. The lean mass remained constant in ACE_i_ but increased (*p* < 0.001) by 3 [2; 4] % in PLA. In healthy middle‐aged adults, 8 weeks of high‐intensity exercise training increases maximal oxygen uptake and skeletal muscle endurance irrespective of ACE inhibitor treatment. However, ACE inhibitor treatment counteracts exercise training‐induced increases in lean mass and left atrial volume. ACE inhibitor treatment compromises total hemoglobin mass.

## Graphical abstract

A high‐intensity endurance exercise training program improves maximal oxygen uptake, whole‐body work capacity, and muscle endurance, with no effect of ACE inhibitor treatment, in normotensive adults. However, ACE inhibitor treatment impairs the exercise‐induced increase in lean mass and left atrial volume and compromises total hemoglobin mass. Results are presented as means with 95% confidence intervals. *p < 0.05, **p < 0.001 for the within‐group change (∆) from baseline from a paired samples t‐test, and #*p* < 0.05 for the between‐group difference in change from baseline from an independent samples t‐test in participants treated with an angiotensin‐converting enzyme inhibitor (ACE_i_) or placebo. CMR, cardiovascular magnetic resonance imaging.

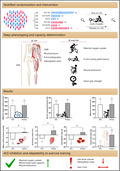

## 1 INTRODUCTION

Angiotensin‐converting enzyme (ACE) is abundantly expressed in pulmonary endothelial cells and catalyzes the conversion of angiotensin I to angiotensin II, which causes general vasoconstriction through the angiotensin type‐1 receptor. ACE also breaks down the key vasodilatory peptide bradykinin (Yang et al., 1970). Marked individual differences in plasma ACE activity are partly explained by the ACE insertion (I)/deletion (D) polymorphism (Rigat et al., 1990). In the late 1990s, the ACE I/D polymorphism became the first genetic element shown to substantially impact human exercise training adaptation (Montgomery et al., 1998). In brief, ACE I/I homozygotes experienced an 11‐fold greater exercise training‐induced improvement in repetitive elbow flexion duration than ACE D/D homozygotes. Since then, the ACE I/I polymorphism has been associated with more marked training‐induced increases in endurance performance, while the ACE D/D polymorphism may increase the potential for strength and power performance (Bray et al., 2009; Li et al., 2017; Ma et al., 2013; Puthucheary et al., 2011; Williams et al., 2017). The underlying cause for ACE genotype to interact with the response to training can be hypothesized to be related to the genotype‐associated difference in ACE activity, but this remains to be determined.

To determine if plasma ACE activity is a determinant for exercise capacity and adaptability, ACE inhibitors appear as a useful tool. ACE inhibitors are widely prescribed to prevent and treat cardiovascular diseases (Mahmoudpour et al., 2018), for which the global prevalence has nearly doubled from 1990 to 532 million cases in 2019 (Roth et al., 2020). Exercise training is another widely applied treatment for cardiovascular diseases because blood pressure is lowered (Borjesson et al., 2016) and all‐cause mortality and cardiovascular disease events are reduced (Kodama et al., 2009). ACE inhibitors reduce circulating angiotensin II and increase plasma bradykinin concentration (Su et al., 1999). Angiotensin type‐1 receptor and bradykinin receptor B2 are widely expressed (Deminice et al., 2020; Figueroa et al., 1996; Matsumoto et al., 2000; Minshall et al., 1995), and their activation partially mediates adaptations to exercise training (Barauna et al., 2008; Massidda et al., 2014). Therefore, ACE inhibitor treatment may affect the general adaptation to exercise training. In support, ACE inhibitor treatment alone has been demonstrated to improve 6‐min walking distance in elderly people with functional impairment (Sumukadas et al., 2007) and heart failure (Hutcheon et al., 2002). In addition, (Buford et al., 2012) reported more pronounced training‐induced improvements in walking speed and mobility in older individuals with mild to moderate functional impairment receiving ACE inhibitors compared to others. However, more recent studies have failed to observe any synergistic effects between ACE inhibitor treatment and exercise training outcomes in functionally impaired elderly (Sumukadas et al., 2014) and hypertensive elderly (Baptista et al., 2018). Moreover, chronic treatment with ACE inhibitors may even reduce aerobic endurance in hypertensive older adults (Baptista et al., 2018), while others did not find any association between ACE inhibitor treatment and markers of physical performance (Cesari et al., 2010; Spira et al., 2016). In rodents, improvements in performance with ACE inhibitors in combination with exercise but not with ACE inhibitors alone have been reported (Habouzit et al., 2009), but conflicting results exist (Minami et al., 2007).

It is clear that existing studies yield conflicting results concerning the importance of ACE activity on functional capacity and adaptation to exercise training. Thus, a randomized controlled study of normotensive untrained participants, including physiological evaluations, is required. The aim of the present study is to investigate the effect of ACE inhibitor treatment on human adaptation to systematic supervised exercise training in a healthy population without confounding diseases and treatments.

## 2 METHODS

### 2.1 Participants

**FIGURE 1:**
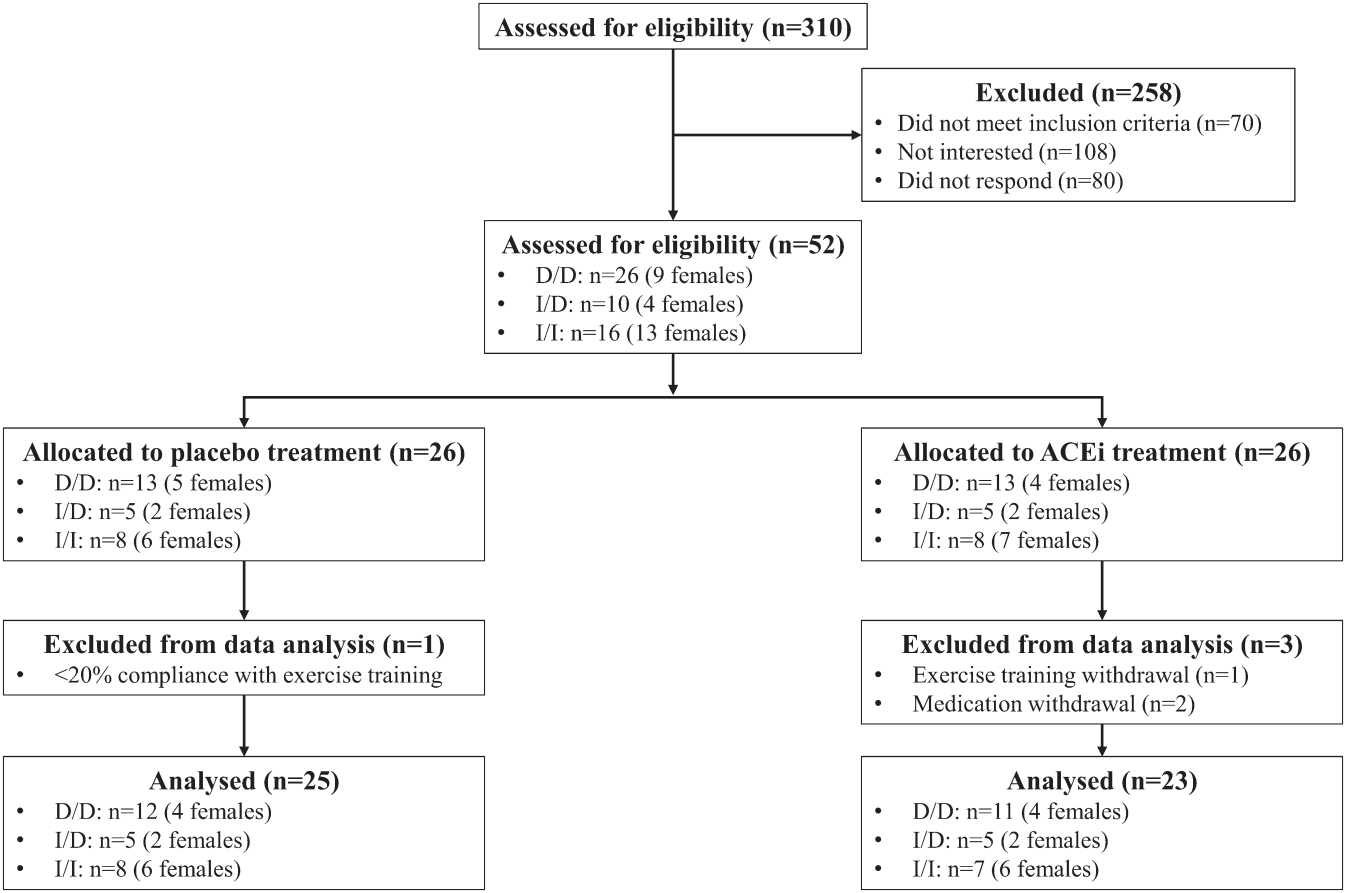
Consort diagram. Stratified randomization by gender and angiotensin‐converting enzyme genotype (D/D, I/D, and I/I) was applied to allocate the study participants to angiotensin‐converting enzyme inhibitor (ACEi) or placebo treatment.

Fifty‐two healthy Faroese participants (26 females, 26 males) provided written informed consent after receiving written and oral information. Participants were included in the study if they were aged 20–50 years and healthy, with no signs of cardiovascular disease. Recruitment occurred through the FarGen‐Infrastructure in the Faroese health care system from May 2019 to July 2019, and the intervention ran from September 2019 to December 2019. Forty‐eight participants completed the intervention (24 females and 24 males; Table 1); see Figure 1 for the study consort diagram. The Faroese and Danish Ethics Committees (H‐18016341) approved the study protocol, which was in accordance with the Declaration of Helsinki. The study was registered at ClinicalTrials.gov (Identifier: NCT03949075), and the study record was first available on ClinicalTrials.gov on 14/05/2019.

**TABLE 1:** Participant characteristics

Variable	Gender	ACE_i_	Placebo
Age (Years)	Male	39 ± 7	39 ± 8
	Female	43 ± 6	41 ± 7
Height (m)	Male	1.79 ± 0.04	1.78 ± 0.04
	Female	1.64 ± 0.06	1.65 ± 0.04
Weight (kg)	Male	87 ± 13	86 ± 12
	Female	74 ± 10	76 ± 13
BMI (kg/m^2^)	Male	27 ± 4	27 ± 4
	Female	28 ± 3	28 ± 5

*Note*: Values are presented as means ± SD.

Abbreviation: ACE_i_, angiotensin‐converting enzyme inhibitor.

### 2.2 Sample size

Cardiovascular fitness, defined as maximal oxygen uptake (VO_2max_), correlates closely with the relative risk for cardiovascular disease (Kodama et al., 2009) and is highly malleable with exercise training. Likewise, muscle phenotype is markedly improved in a health‐beneficial direction by exercise training (McGee & Hargreaves, 2020). Therefore, VO_2max_ and muscle endurance were selected as primary outcomes in the present study. The sample size was calculated to detect changes comparable to previous exercise studies (Børve et al., 2017; Leon et al., 2000). A between‐group difference of >20% in the adaptation of the primary outcomes was considered clinically relevant. At a power of 0.8 and a *p* = 0.05 level of significance, 23 participants per group were required.

### 2.3 ACE genotype

ACE genotype was determined to ensure a balanced genotype frequency in the experimental groups. Primer design, amplification, and detection of the ACE I/D polymorphism have been described previously (Lin et al., 2001). Briefly, for real‐time amplification and detection of the ACE polymorphism, SYBR Green I PCR Master Mix reagents (Applied Biosystems) were used on the StepOnePlus™ Real‐Time PCR System (Applied Biosystems) in combination with a melting curve analysis.

### 2.4 Experimental design

A randomized, double‐blind, placebo‐controlled design was applied. Participants were stratified by gender and ACE genotype to ingest either placebo (PLA: CaCO_3_) or an ACE inhibitor (ACE_i_: Enalapril, Krka, Novo mesto, Slovenia) for 8 weeks while following a supervised exercise training program. ACE inhibitor and placebo administration were initiated on the first day of exercise. The stratified randomization was generated by a random number generator in SPSS (IBM SPSS Statistics, version 27.0.0), which included the variables subject ID, gender, ACE I/D genotype, and group (ACE_i_ and PLA). Randomization was based on a fixed block size to ensure pre‐specified group size. A physician not participating in experimental trials or data analysis performed the randomization. The study was double‐blinded with regard to ACE genotype and study medication, and the blinding was kept until the completion of the final clinical investigation. A deep‐phenotypical characterization was conducted <3 weeks before and <5 days post‐intervention at the Research Park iNOVA and the National hospital of the Faroe Islands.

Enalapril dosage was increased gradually from 5 mg/d on day 2 to 10 mg/d, 15 mg/d, and 20 mg/d on days 5, 8, and 11, respectively. A maximum of 10 mg ACE inhibitor was taken in the morning and evening to minimize the risk of side effects (Izzo & Weir, 2011). For safety, all participants monitored and reported resting blood pressure twice daily using a home‐based device (IM101 Intermedico, Hellerup, Denmark) during Weeks 1 and 2. Treatment dosage was reduced by 50%–75% if intolerable side effects occurred. Intolerable side effects were reported by five females and three males in ACE_i_ and four females in PLA. Side effects included dizziness, fatigue, malaise, and blurry vision.

### 2.5 Exercise training

Participants completed supervised ergometer rowing (Concept 2 model D w. PM5, VT, USA) sessions lasting ~1 h, three times weekly for 8 weeks. Rowing was chosen to engage both upper and lower body musculature while inducing a marked cardio‐respiratory and metabolic strain. Power output was registered (Table 2).

**TABLE 2:** Training volume and intensity

Training	Treatment	Active time	Average power output	Average relative power output
Warm‐up	ACE_i_	6 min/session	128 ± 36 W	85% ± 17%
	PLA		129 ± 32 W	86% ± 18%
Intervals	ACE_i_	16 min/session	169 ± 49 W	111% ± 11%
	PLA		175 ± 46 W	114% ± 14%
Cool down^1^	ACE_i_	5 min/session	113 ± 33 W	75% ± 19%
	PLA		113 ± 28 W	75% ± 17%

*Note*: Values are presented as means ± SD. ^1^Cool down was included in 12 sessions. The average relative power output is the average power output normalized to the average power participants could sustain during a 6 min all‐out rowing‐ergometer effort performed prior to the first training session.

Abbreviation: ACE_i_, angiotensin‐converting enzyme inhibitor.

Efficient high‐intensity interval training was applied to improve cardiovascular capacity (MacInnis & Gibala, 2017). Training consisted predominantly (~70%) of short‐duration (≤4 min) exhaustive high‐intensity bouts, utilizing a 1:1 work‐to‐rest ratio. Training adherence was 99.9% (range 96%–100%).

### 2.6 Deep phenotype characterization

Before and after the eight‐week intervention, each participant underwent an extensive evaluation of training‐sensitive physiological parameters. Participants reported to the laboratory in a post‐absorptive state (<3 h) and refrained from vigorous exercise 24 h before all assessments. Alcohol, tobacco, and caffeine were avoided on the evaluation days.

#### 2.6.1 Exercise capacity

Exercise capacity can be determined in several ways. VO_2max_ determined by stepwise increased workload on a cycle ergometer is often used (Snell et al., 2007). Additionally, the capacity to complete physical work with a large active muscle mass (e.g., rowing) in a given time frame (e.g., 6 min) is the result of both VO_2max_ and the ability to sustain a high percentage of VO_2max_, which is determined by muscular endurance (Joyner & Coyle, 2008). Finally, muscle group‐specific endurance, independent of cardiorespiratory fitness, can be evaluated by engaging a limited muscle mass in an endurance exercise protocol, where the results are highly dependent on local muscular oxidative capacity and muscle mass (Saltin et al., 1976). All three aspects of exercise capacity were evaluated in the present study. Furthermore, maximal voluntary contraction force was assessed using a handgrip dynamometer.

Firstly, maximal voluntary handgrip contraction force was determined in a fixed seated position. After 6 min light squeezing of a JAMAR hand dynamometer (Performance Health), three maximal 3 s contractions were completed, separated by 30 s of rest. The maximal value was recorded.

Following 5 min rest, skeletal muscle endurance was assessed. Participants pulled a handle from a stretched arm position to a 90‐degree elbow flexion (Sygnum Plate‐Loaded Seated Rowing Machine, Gym80, Germany) in a consistent 60 bpm rhythm until exhaustion, and the time was recorded. Resistance was set at ~40% of maximal voluntary elbow flexion.

After 15 min of rest, VO_2max_ and cycling (Excalibur Sport, Lode, Groningen, Netherlands) peak workload (W_max_) were determined. Warm‐up for 4 min at 40 or 50 W and 6 min at 80 or 100 W for females and males, respectively, was followed by a workload increase of 20 (females) or 25 (males) W/min until exhaustion. W_max_ was noted. Oxygen uptake (Cosmed, Quark b2, Milan, Italy) and heart rate (HRM‐Dual, Garmin, Olathe, KS, USA) were measured continuously. VO_2max_ was defined as the highest 30 s average recorded.

On a separate day, a 6 min all‐out rowing‐ergometer effort was completed, and mean power output was recorded.

The exercise capacity assessments were organized in the order mentioned above to minimize the impact on each other. The recovery time for maximal force generation following ≤3 maximal isometric contractions is less than 5 min (Watanabe et al., 2005). As for the upper‐body skeletal muscle endurance assessment, the involved muscle groups were arguably not fully recovered for the subsequent assessment of whole‐body VO_2max_. However, the direct impact of upper‐body musculature fatigue on the assessment of VO_2max_ was expected to be negligible, because VO_2max_ was evaluated during cycling. In addition, the 15 min recovery between the assessments should be sufficient to revert the majority of metabolic disturbances (i.e. blood lactate, pH, and HCO_3−_). Following the evaluation of VO_2max_, severe fatigue must be expected, and therefore the 6 min all‐out rowing test was completed on a separate day.

#### 2.6.2 Blood pressure

Blood pressure was measured two times 1 min apart and in accordance with the American Heart Association's guidelines (Pickering et al., 2008). Briefly, after ≥5 min of rest, blood pressure was measured in a seated position with the back supported and the feet flat on the floor. The arm was supported on a flat surface, with the upper arm at heart level and the bottom of the cuff directly above the elbow bend.

#### 2.6.3 Hemoglobin mass and blood volume

Hemoglobin mass is one of several determinants of cardiovascular capacity (Montero & Lundby, 2019). Total hemoglobin mass was determined by carbon monoxide rebreathing as previously described (Schmidt & Prommer, 2005). Based on the total hemoglobin mass, calculations of blood volume (total hemoglobin mass [g]/hemoglobin concentration [g/L]), red cell volume (blood volume × hematocrit), and plasma volume (blood volume – red cell volume) were performed.

#### 2.6.4 Cardiovascular magnetic resonance imaging

Cardiac dimensions and function closely reflect exercise capacity and are highly responsive to exercise training (Arbab‐Zadeh et al., 2014; Fujimoto et al., 2010). Cardiac images were obtained on a 1.5‐Tesla MRI scanner equipped with a 16‐channel cardiac coil and sequences gated to the ECG (Aera, Siemens Medical Solutions). Multiple short‐axis cine covering the entire cardiac fossa were obtained (SSFP, thickness 8 mm, no gap, echo time 1.5 ms, repetition‐time 3.0–3.2 ms; flip‐angle 60°; 192 × 192 matrix; parallel imaging technique and 25 phases). Cardiac volumes were determined by an experienced medical doctor using semi‐automated software (CVi42, v5.2.1, Circle Cardiovascular Imaging Inc., 11) (Kyhl et al., 2013). End‐diastolic and end‐systolic volume were defined as the maximum and minimum ventricular volume, respectively. Stroke volume was the difference between end‐diastolic and end‐systolic volume. Ejection fraction was stroke volume divided by end‐diastolic volume (%).

#### 2.6.5 Echocardiography

Echocardiography was performed using Vivid E9 ultrasound systems (GE Healthcare) with a S5, 2.5‐MHz transducer by an experienced medical doctor. All echocardiograms were analyzed using dedicated software (EchoPac, GE Medical). A 2D and color TDI echocardiography was applied. In the parasternal long‐axis view, 2‐dimensional images were used to quantify the myocardial thickness and the dimensions of the left ventricle. Evaluation of left ventricular ejection fraction was calculated using the modified Simpson's rule. The mitral inflow velocity was measured using pulsed‐wave Doppler in the apical 4‐chamber position at the tips of the mitral valve leaflets and peak velocity of early (E) and atrial (A) diastolic filling and deceleration time of the E‐wave (DT) were measured. In the apical 4‐chamber position, color TDI tracings with the range gate at the septal and lateral mitral annular were acquired to determine the peak longitudinal early diastolic (eʹ) velocity as the average between lateral and septal velocities. The left atrial size was determined from apical 4‐ and 2‐chamber images. M‐mode of the tricuspid annular systolic motion was performed to access right ventricular function.

#### 2.6.6 Body composition

Total body fat and lean tissue was assessed using dual‐energy x‐ray absorptiometry (DXA) (Norland XR‐800, Norland Corporation).

### 2.7 Statistics

Data are presented as means and 95% confidence intervals unless otherwise stated. Continuous endpoints were compared between treatment groups by a mixed‐model repeated‐measures approach using the SPSS MIXED procedure (Cnaan et al., 1997). The analysis included treatment (ACE_i_ vs. PLA), time (before vs. after intervention), and a time×treatment interaction as fixed effects. The difference in response between treatment groups was assessed by the interaction effect. Repeated measures and random factor was defined from individual participants. A significant effect of time or interaction was further evaluated by a Sidak‐adjusted pairwise comparison.

The independence of the obtained data was assumed in the model. All data were subjected to visual inspection of normality of the residuals and homogeneity of residual variance. Data outside the mean ± 3 × standard deviations were excluded, resulting in the omission of one left atrial volume data point in PLA. The level of statistical significance was set to *p* < 0.05. SPSS was used for statistical analyses (IBM SPSS Statistics, version 27.0.0).

## 3 RESULTS

PLA and ACE_i_ had similar gender and ACE genotype distribution (Figure 1) and were comparable in age, height, weight, and body mass index (Table 1). Except for left atrial volume, all outcome measures were similar in the two groups at baseline.

### 3.1 Exercise capacity

**FIGURE 2:**
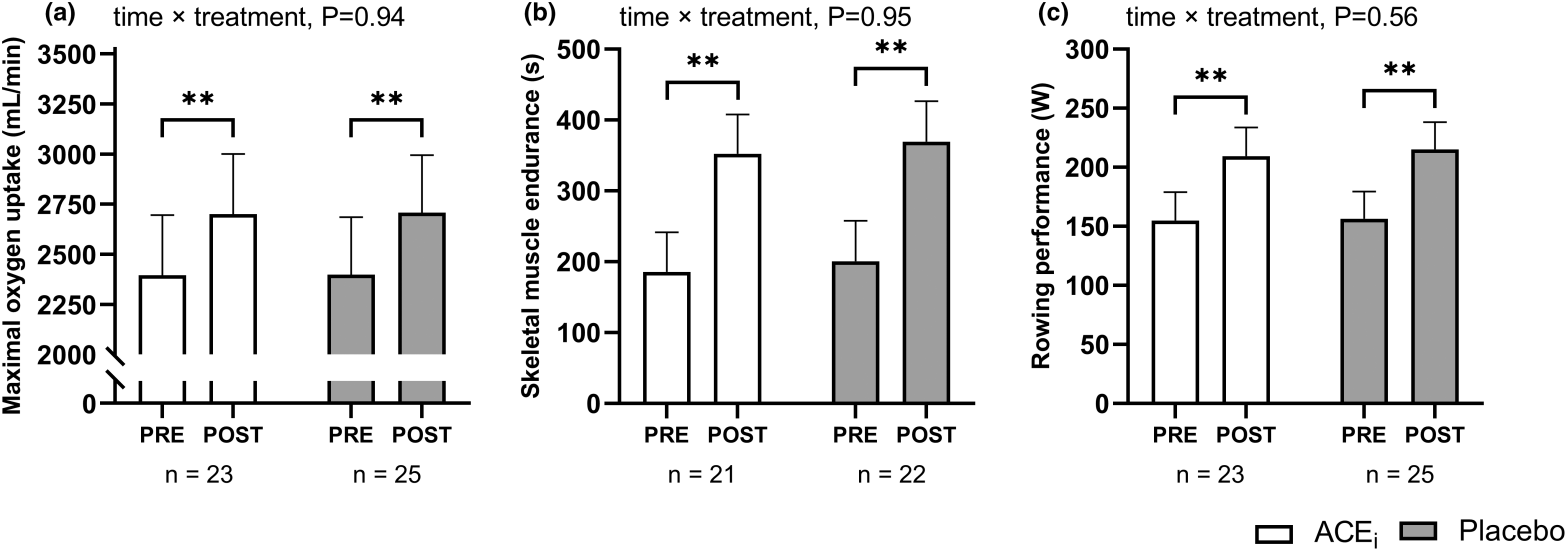
Exercise capacity adaptations. Values are presented as means (with 95% confidence intervals) from a linear mixed‐model with time, treatment, and time × treatment as explanatory variables. The figure shows maximal oxygen uptake (a), skeletal muscle endurance (b), and rowing performance (c) measured pre‐and post‐intervention in participants treated with an angiotensin‐converting enzyme inhibitor (ACE_i_ ) or placebo. If a significant effect of time × treatment or time existed, the result of the post hoc analysis is indicated by *p < 0.05, **p < 0.001 compared with pre‐intervention.

VO_2max_ and upper‐body muscle endurance increased (*p* < 0.001) similarly in both groups by 304 [201; 408] ml/min and 82 [55; 113] %, respectively, in ACE_i_ and by 310 [210; 409] ml/min and 74 [48; 105] %, respectively, in PLA (Figure 2a,b). Both groups also demonstrated similar improvements (*p* < 0.001) in rowing performance, with magnitudes of 55 [44; 65] W in ACE_i_ and 59 [49; 69] W in PLA (Figure 2c), and in cycling W_max_, with magnitudes of 21 [16; 26] W (*n* = 23) in ACE_i_ and 26 [21; 31] W (*n* = 25) in PLA. Maximal isometric hand‐grip strength remained unchanged in both ACE_i_ (0.0 [−1.7; 1.7] kg [*n* = 23]) and PLA (1.2 [−0.4; 2.8] kg [*n* = 25]).

### 3.2 Blood pressure

**FIGURE 3:**
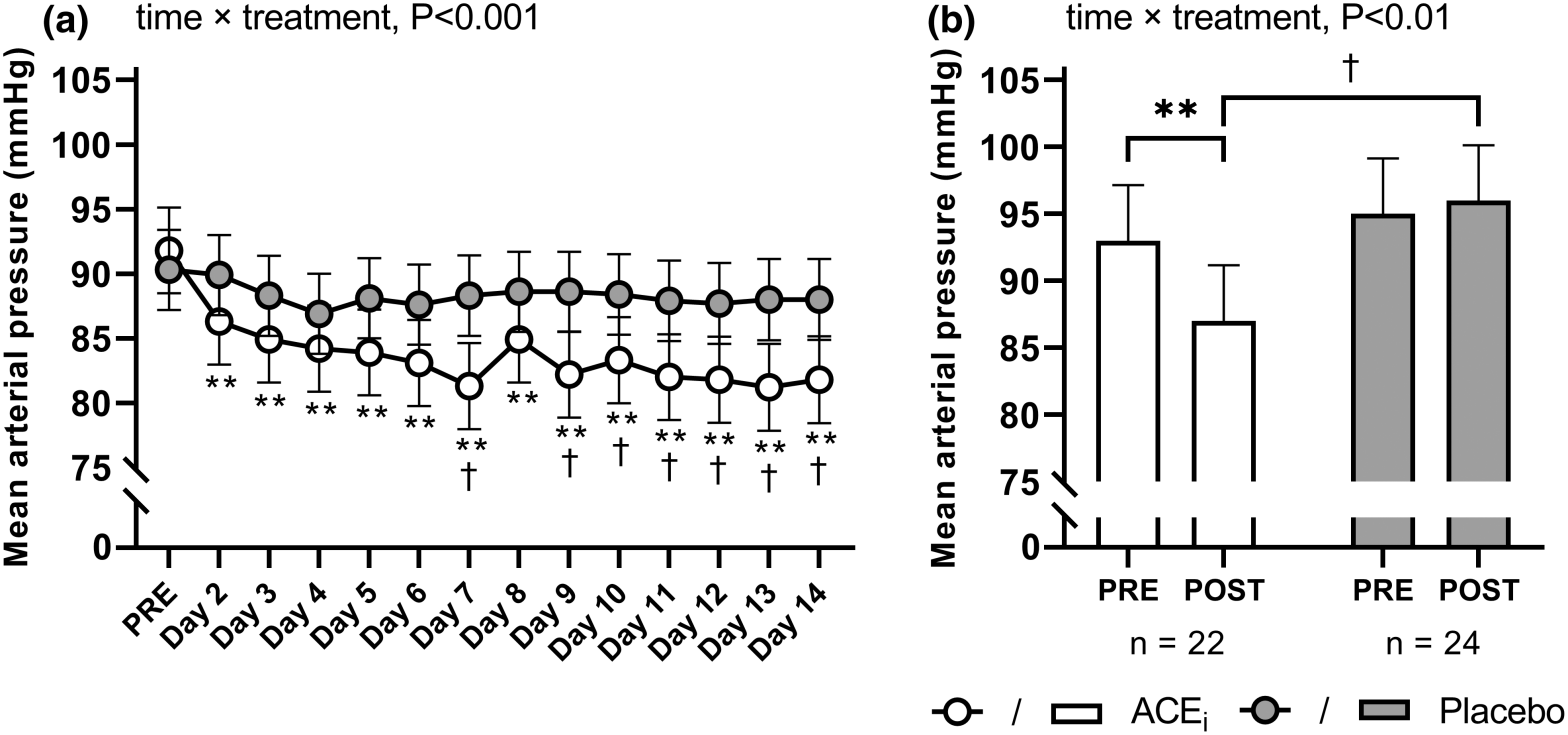
Treatment efficiency. Values are presented as means (with 95% confidence intervals) from a linear mixed‐model with time, treatment, and time × treatment as explanatory variables. The figure shows mean arterial blood pressure measured at home during the first 2 weeks of the intervention (a) and mean arterial blood pressure measured in a clinical setting pre‐and post‐intervention (b) in participants treated with an angiotensin‐converting enzyme inhibitor (ACE_i_ ) or placebo. If a significant effect of time × treatment or time existed, the result of the post hoc analysis is indicated by **p < 0.001 compared with pre‐intervention, and ^†^ *p* < 0.05 compared with placebo.

Home‐based mean arterial pressure (MAP) recordings were reduced in ACE_i_ from day two and onwards, while no changes were observed in PLA (time × treatment: *p* < 0.001; Figure 3a). Likewise, laboratory determined MAP measurements were reduced (*p* < 0.001) by −5.5 [−8.4; −2.6] mmHg from pre‐ to post‐intervention in ACE_i_, whereas no changes were apparent in PLA (time × treatment: *p* < 0.01; Figure 3b).

### 3.3 Cardiovascular characteristics

**FIGURE 4:**
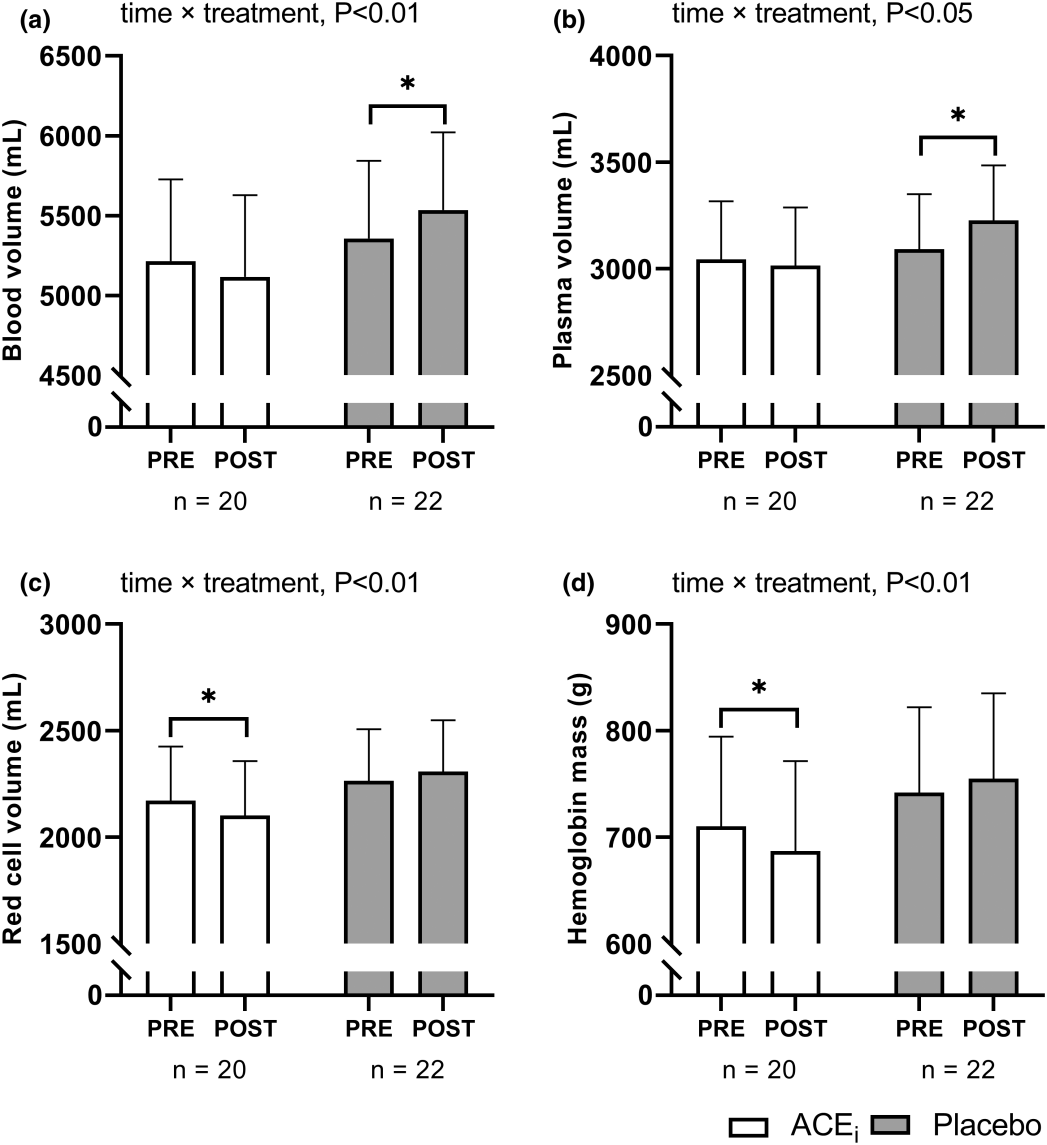
Hematological adaptations. Values are presented as means (with 95% confidence intervals) from a linear mixed‐model with time, treatment, and time × treatment as explanatory variables. The figure shows total blood volume (a), plasma volume (b), hemoglobin mass (c), and red cell volume (d) measured pre‐and post‐intervention in participants treated with an angiotensin‐converting enzyme inhibitor (ACEi) or placebo. If a significant effect of time × treatment or time existed, the result of the post hoc analysis is indicated by *p < 0.05 compared with pre‐intervention.

A time×treatment interaction (*p* < 0.05) existed for all obtained hematological measurements (Figure 4). In PLA, exercise training increased (*p* < 0.05) total blood volume by 178 [41; 314] ml (Figure 4a), which was primarily related to the 136 [30; 241] ml plasma volume expansion (*p* < 0.05) (Figure 4b) as red cell volume and hemoglobin mass was unaffected (Figure 4c,d). In contrast, blood volume and plasma volume did not increase in ACE_i_, but a reduction (*p* < 0.05) in total hemoglobin mass of −23 [−40; −6] g and red cell volume of −69 [−121; −17] ml existed.

In addition, the applied intervention affected diastolic function (Table 3). Left atrial volume was increased (*p* < 0.01) by 5.5 [1.9; 9.0] ml in PLA and decreased (*p* < 0.05) by −4.2 [−7.6; −0.7] ml in ACE_i_ (time × treatment: *p* < 0.01; Table 3). In addition, mitral valve deceleration time velocity remained unaltered in PLA but decreased (*p* < 0.05) by −28.1 [−50.1; −6.1] ms in ACE_i_ (time × treatment: *p* < 0.01; Table 3).

**TABLE 3:** Cardiac characteristics

	ACE_i_			Placebo			
Outcome	pre	post	*n*	pre	post	*n*	time × treatment
Cardiac remodeling assessed by cardiac magnetic resonance imaging							
LVEDV (ml)	150 [138; 163]	146 [134; 159]	23	148 [136; 160]	150 [138; 163]	25	*p* = 0.05
LVSV (ml)	91.7 [83.8; 99.7]	91.3 [83.3; 99.2]	23	89.8 [82.2; 97.5]	92.5 [84.9; 100.2]	25	*p* = 0.25
LVEF (%)	61.2 [59.0; 63.5]	62.8 [60.5; 65.0]	23	60.9 [58.8; 63.1]	61.4 [59.3; 63.6]	25	*p* = 0.51
LV mass (g)	127 [116; 139]	126 [115; 138]	23	131 [120; 142]	133 [122; 144]	24	*p* = 0.24
Diastolic function assessed by echocardiography							
LAV (ml)	46.9 [42.6; 51.1]†	42.7 [38.5; 46.9]*	21	40.1 [35.7; 44.4]	45.5 [41.2; 49.9]*	20	*p* < 0.01
MV E/A	1.30 [1.15; 1.46]	1.44 [1.29; 1.59]	21	1.35 [1.21; 1.50]	1.30 [1.15; 1.44]	23	*p* = 0.06
MVDT (ms)	198 [178; 217]	170 [150; 189]*†	21	191 [172; 209]	208 [190; 227]	23	*p* < 0.01
e′ (cm/s)	12.76 [11.86; 13.67]	12.69 [11.78; 13.60]	21	12.72 [11.85; 13.58]	12.37 [11.50; 13.24]	23	*p* = 0.57
MV E/e′	6.5 [5.8; 7.3]	7.0 [6.3; 7.7]	21	6.5 [5.8; 7.1]	6.9 [6.2; 7.6]	23	*p* = 0.98
TRmax (mmHg)	22.1 [20.0; 24.1]	21.1 [19.1; 23.1]	14	19.5 [17.6; 21.5]	20.9 [18.9; 22.8]	15	*p* = 0.09

*Note*: Values are presented as means (with 95% confidence intervals) from a linear mixed‐model with time, treatment, and time × treatment as explanatory variables. If a significant effect of time × treatment or time existed, the result of the post hoc analysis is indicated by *p < 0.05 compared with pre‐intervention, and ^†^ *p* < 0.05 compared with placebo. Abbreviations: ACE_i_, angiotensin‐converting enzyme inhibitor; eʹ, early diastolic mitral annulus velocity; LAV, left atrial volume; LV mass, left ventricular mass; LVEDV left ventricular end‐diastolic volume; LVEF, left ventricular ejection fraction; LVSV, left ventricular stroke volume; MV E/A, mitral valve E/A ratio; MV E/e′, mitral valve E/é ratio; MVDT, mitral valve deceleration time; TRmax, tricuspid regurgitation peak velocity.

### 3.4 Body composition

**FIGURE 5:**
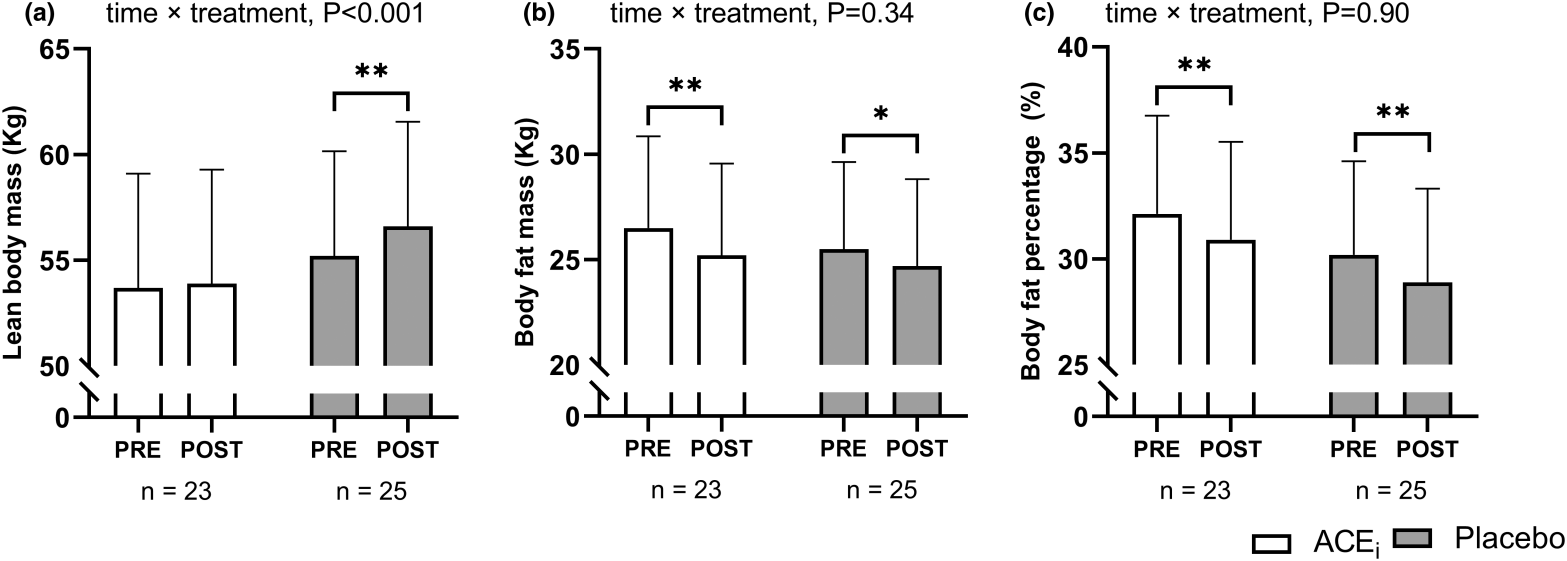
Body composition adaptations. Values are presented as means (with 95% confidence intervals) from a linear mixed‐model with time, treatment, and time × treatment as explanatory variables. The figure shows lean body mass (a), body fat mass (b), and body fat percentage (c) measured pre‐and post‐intervention in participants treated with an angiotensin‐converting enzyme inhibitor (ACE_i_ ) or placebo. If a significant effect of time × treatment or time existed, the result of the post hoc analysis is indicated by *p < 0.05, **p < 0.001 compared with pre‐intervention.

Exercise training increased (*p* < 0.01) lean body mass by 1.5 [1.0; 2.0] kg in PLA, whereas it was unaffected in ACE_i_ (time × treatment: *p* < 0.01; Figure 5a). However, body fat mass was reduced similarly in both groups by −1.4 [−2.1; −0.6] kg (*p* < 0.001) in ACE_i_ and − 0.9 [−1.6; −0.2] kg (*p* < 0.05) in PLA (Figure 5b). In addition, both groups reduced body fat percentage to a similar extent (Figure 5c).

## 4 DISCUSSION

The present randomized, double‐blind, placebo‐controlled trial determined whether exercise adaptations are affected by concurrent ACE inhibitor treatment. Training‐induced adaptations in exercise capacity occurred, to a large extent, independently of ACE inhibitor treatment. However, ACE inhibitor treatment impaired the exercise‐induced increase in lean mass and left atrial volume and caused a reduction in hemoglobin mass. These novel findings are relevant in patients treated with ACE inhibitors and further add to understanding the role of the renin‐angiotensin‐aldosterone system in driving adaptation to exercise training.

### 4.1 Cardiovascular adaptations

Exercise capacity, determined as cardio‐respiratory fitness or whole‐body VO_2max_, is a robust predictor of all‐cause mortality and cardiovascular disease (Kodama et al., 2009). In the present study, all measures of exercise capacity increased similarly in both groups. VO_2max_ increased by ~13%, corresponding to a ~ 1‐MET higher level of maximal aerobic capacity, which is associated with a 13% lower risk of all‐cause mortality and a 15% lower risk of cardiovascular disease events in healthy individuals (Kodama et al., 2009). Prior evidence demonstrating a positive impact of ACE inhibitor treatment alone on markers of exercise capacity (Hutcheon et al., 2002; Sumukadas et al., 2007) has led to more recent investigations of the potential additive effects of ACE inhibition on exercise adaptability (Baptista et al., 2018; Buford et al., 2012; Habouzit et al., 2009; Sumukadas et al., 2014). In accordance with our findings, (Sumukadas et al., 2014) reported no synergistic effect of ACE inhibitor treatment on training‐induced improvements in exercise capacity in functionally impaired elderly. Similar observations have recently been replicated in hypertensive elderly (Baptista et al., 2018). In contrast, (Buford et al., 2012) reported enhanced exercise responsiveness in walking speed and chair‐rise ability in elderly ACE inhibitor users compared to users of other antihypertensive drugs or nonusers. These contradictory observations should be interpreted carefully as the inclusion of participants with comorbidities, and the concomitant use of various medical treatments (Buford et al., 2012; Sumukadas et al., 2014), as well as the use of different types of ACE inhibitors (Baptista et al., 2018), might potentially mask the isolated effect of ACE inhibitor treatments on exercise adaptability. These confounding factors were controlled for in the present study by administrating mono‐dose enalapril treatment to a healthy population without confounding diseases and medical treatments. Our results confirm that ACE inhibitor treatment does not impact adaptations in exercise capacity after a period of high‐intensity training. In addition, previous studies assessing the impact of ACE inhibitor treatment on exercise adaptations have not controlled for the ACE I/D genotype, which has been strongly associated with plasma ACE activity (Rigat et al., 1990) and purported to play a significant role in exercise capacity adaptations (Bray et al., 2009; Puthucheary et al., 2011). The present study determined the ACE I/D genotype to ensure a balanced ACE II/ID/DD genotype frequency in the treatment groups. To assess the possible isolated effect of ACE I/D genotype on exercise capacity adaptations, a sub‐analysis constituting ACE genotype, time, and a time × genotype interaction as fixed effects was completed. However, no interaction was detected from the obtained markers of exercise capacity (data not shown). Similarly, no time × genotype × treatment interaction was evident, indicating no confounding effect of the ACE I/D genotype on the interaction between ACE inhibitor treatment and exercise capacity adaptations (data not shown).

Improved aerobic capacity following ~6 weeks of endurance training arguably relates to an increased hemoglobin mass and red cell volume in healthy untrained volunteers (Montero & Lundby, 2019). However, the reduced hemoglobin mass after ACE inhibitor treatment in the present study demonstrates that VO_2max_ and exercise capacity can improve even with a concomitant red cell volume reduction. The reduction in hemoglobin mass with exercise training and ACE_i_ treatment may appear surprising. However, low‐dose (5 mg/day) Enalapril treatment reduces plasma erythropoietin ~20% in healthy volunteers (Pratt et al., 1992), and 6 months of Enalapril treatment reduces red blood cell mass in kidney transplant erythrocytosis patients (Perazella et al., 1995), potentially as a result of an angiotensin‐II mediated genetic regulation of erythropoietin synthesis in human kidney cells via angiotensin‐II receptor type 1 (Kim et al., 2014; Montero & Lundby, 2019). Therefore, the current observation likely reflects a balance between Enalapril‐induced inhibition and exercise training induction of erythropoiesis. Thus, the addition of exercise training to ACE inhibitor treatment appears advisable to counteract a potential reduction in blood volume.

In relation to cardiac adaptations to exercise training, we observed a 14% increase in left atrial volume in PLA, whereas a 9% reduction was observed in ACE_i_. Left atrial volume expansion is an expected adaptation to exercise training (Mahjoub et al., 2019), and enlarged chamber dimensions are considered a typical finding of the athlete's heart (Maron & Pelliccia, 2006). In conjunction with the tendency for interaction (time × treatment: *p* = 0.05) for left ventricular end‐diastolic volume to be increased ~1% in PLA and decreased ~2.5% in ACE_i_, the current findings indicate that ACE inhibitor treatment compromises expected exercise training‐induced adaptation in cardiac dimensions. However, the effects are relatively small, and the long‐term effects are unknown. Notably, more pronounced cardiac adaptations must be expected with exercise protocols lasting more than 3 months (Arbab‐Zadeh et al., 2014; Fujimoto et al., 2010). Indeed, 3 months of intensive endurance exercise can induce concentric remodeling, including increased left ventricular mass and wall‐thickness in young, healthy, previously sedentary participants (Arbab‐Zadeh et al., 2014). If training is continued for 12 months, left ventricular dilation and restoration of mass to volume ratio occurs in young (Arbab‐Zadeh et al., 2014) but not necessarily in older individuals (Fujimoto et al., 2010). Thus, the present findings warrant further investigation in a prolonged exercise training and ACE inhibitor treatment trial in relevant patient groups.

### 4.2 Body composition

Lean body mass increased by 1.5 kg in PLA but remained unchanged in ACE_i_. Augmented lean body mass is an important adaptation to exercise training, especially for sedentary and elderly individuals, as it likely reflects an increased muscle mass (Scafoglieri & Clarys, 2018). Since ACE inhibitors are widely prescribed for several lifestyle‐and age‐related cardiovascular indications such as hypertension, heart failure, and myocardial infarction (Mahmoudpour et al., 2018), practitioners must consider the potential negative interaction between treatment with ACE inhibitors and exercise‐induced muscle hypertrophy. Consistent with our findings, ACE inhibitor treatment has been associated with reduced, relative BMI‐adjusted muscle mass in older individuals (Spira et al., 2016). In addition, evidence from animal studies suggests that angiotensin II, which is reduced by ACE inhibitor treatment, is necessary for optimal overload‐induced hypertrophy, most likely mediated through an angiotensin type‐1 receptor‐mediated signaling pathway (Gordon et al., 2001; McBride, 2006; Westerkamp et al., 2005). Moreover, human studies have shown that individuals with an innate disposition towards higher levels of serum ACE and thus higher production of angiotensin II, demonstrate greater strength gains in response to exercise training (Folland et al., 2000; Giaccaglia et al., 2008; Puthucheary et al., 2011). Periodization of ACE inhibitor treatment and strength training may be considered but warrants further investigation. However, it must be noted that the observed between‐group difference in lean body mass adaptation did not manifest in a significant between‐group difference in hand‐grip strength in the present study. In accordance, Spira et al. (2016) reported no negative association between the intake of ACE inhibitors and hand‐grip strength despite the reduced BMI‐adjusted muscle mass in ACE inhibitor users.

### 4.3 Strengths and limitations

The present study has several strengths. Firstly, mean arterial pressure reductions only occurred after ACE inhibitor treatment, demonstrating that the pharmacological intervention was successful. Secondly, the 99.9% adherence to the exercise training intervention is an apparent strength, and marked training‐induced improvements in markers of cardiovascular health and exercise capacity confirm the efficiency of the applied exercise regime. Limitations of the study also exist. The present findings are based on normotensive participants and require patient group‐specific validation. However, the inclusion of healthy participants likely provides the most generally translational observations since patient groups often will be subjected to other pharmacological treatments and frequently exhibit comorbidities.

Moreover, monitoring blood pressure daily at home during the initial 2 weeks of the intervention may have revealed group allocation to participants, as blood pressure would have decreased in the ACE_i_ participants. In an attempt to circumvent this issue, all participants were informed that reduced blood pressure is also an expected outcome of physical training.

## 5 CONCLUSIONS

In conclusion, 8 weeks of high‐intensity physical activity increases whole‐body maximal oxygen uptake and skeletal muscle endurance independent of ACE inhibitor treatment in normotensive adults. However, the administration of ACE inhibitor impairs the exercise‐induced increase in lean mass and left atrial volume and compromises total hemoglobin mass. Consequently, ACE inhibitor treatment can be advised simultaneously with exercise training to improve cardiovascular health, but the implication for lean mass adaptation and red cell mass must be considered. Furthermore, research is needed to examine whether specific patient group considerations are relevant and if treatment periodization may enhance the outcome when ACE inhibitor treatment and exercise training is combined.

## AUTHOR CONTRIBUTIONS

Nikolai B. Nordsborg conceived the study. Nikolai B. Nordsborg, Magni Mohr, Pál Weihe, and Tórur Sjúrðarson finalized the design. All authors performed experiments. Tórur Sjúrðarson, Jacob Bejder, Magni Mohr, and Nikolai B. Nordsborg analyzed data. Tórur Sjúrðarson, Jacob Bejder, Magni Mohr, Kasper Kyhl, and Nikolai B. Nordsborg interpreted the results of the experiments. Tórur Sjúrðarson prepared figures. Tórur Sjúrðarson, Jacob Bejder, and Nikolai B. Nordsborg drafted the manuscript. All authors contributed to writing and reviewing the manuscript and approved the final version of the manuscript.

## FUNDING INFORMATION

The study was supported by the Research Council Faroe Islands (project number 0352) and the Novo Nordisk Foundation (NNF17OC0029134). Moreover, Nikolai B. Nordsborg and Thomas Bonne were partly supported by The Novo Nordisk Foundation grant to Team Danmark.

## CONFLICT OF INTEREST

None. The results of the present study are presented clearly, honestly, and without fabrication, falsification, or inappropriate data manipulation.

## References

[phy215382-bib-0001] Arbab‐Zadeh, A. , Perhonen, M. , Howden, E. , Peshock, R. M. , Zhang, R. , Adams‐Huet, B. , Haykowsky, M. J. , & Levine, B. D. (2014). Cardiac remodeling in response to 1 year of intensive endurance training. Circulation, 130(24), 2152–2161. 25281664 10.1161/CIRCULATIONAHA.114.010775 PMC5698012

[phy215382-bib-0002] Baptista, L. C. , Machado‐Rodrigues, A. M. , Veríssimo, M. T. , & Martins, R. A. (2018). Exercise training improves functional status in hypertensive older adults under angiotensin converting enzymes inhibitors medication. Experimental Gerontology, 109, 82–89. 28645696 10.1016/j.exger.2017.06.013

[phy215382-bib-0003] Barauna, V. G. , Magalhaes, F. C. , Krieger, J. E. , & De Oliveira, E. M. (2008). AT1 receptor participates in the cardiac hypertrophy induced by resistance training in rats. American Journal of Physiology. Regulatory, Integrative and Comparative Physiology, 295(2), 381–387. 10.1152/ajpregu.00933.2007 18495827

[phy215382-bib-0004] Borjesson, M. , Onerup, A. , Lundqvist, S. , & Dahlof, B. (2016). Physical activity and exercise lower blood pressure in individuals with hypertension: Narrative review of 27 RCTs. British Journal of Sports Medicine, 50(6), 356–361. 26787705 10.1136/bjsports-2015-095786

[phy215382-bib-0005] Børve, J. , Jevne, S. N. , Rud, B. , & Losnegard, T. (2017). Upper‐body muscular endurance training improves performance following 50 min of double poling in well‐trained cross‐country skiers. Frontiers in Physiology, 8, 690. 29018351 10.3389/fphys.2017.00690 PMC5615216

[phy215382-bib-0006] Bray, M. S. , Hagberg, J. M. , Pérusse, L. , Rankinen, T. , Roth, S. M. , Wolfarth, B. , & Bouchard, C. (2009). The human gene map for performance and health‐related fitness phenotypes: The 2006–2007 update. Medicine and Science in Sports and Exercise, 41(1), 34–72. 10.1249/mss.0b013e3181844179 19123262

[phy215382-bib-0007] Buford, T. W. , Manini, T. M. , Hsu, F. C. , Cesari, M. , Anton, S. D. , Nayfield, S. , Stafford, R. S. , Church, T. S. , Pahor, M. , & Carter, C. S. (2012). Angiotensin‐converting enzyme inhibitor use by older adults is associated with greater functional responses to exercise. Journal of the American Geriatrics Society, 60(7), 1244–1252. 22726232 10.1111/j.1532-5415.2012.04045.x PMC3625953

[phy215382-bib-0008] Cesari, M. , Pedone, C. , Antonelli Incalzi, R. , & Pahor, M. (2010). ACE‐inhibition and physical function: Results from the trial of angiotensin‐converting enzyme inhibition and novel cardiovascular risk factors (TRAIN) study. Journal of the American Medical Directors Association, 11(1), 26–32. 20129212 10.1016/j.jamda.2009.09.014 PMC2818218

[phy215382-bib-0009] Cnaan, A. , Laird, N. M. , & Slasor, P. (1997). Using the general linear mixed model to analyse unbalanced repeated measures and longitudinal data. Statistics in Medicine, 16(20), 2349–2380. 9351170 10.1002/(sici)1097-0258(19971030)16:20<2349::aid-sim667>3.0.co;2-e

[phy215382-bib-0010] Deminice, R. , Hyatt, H. , Yoshihara, T. , Ozdemir, M. , Nguyen, B. , Levine, S. , & Powers, S. (2020). Human and rodent skeletal muscles express angiotensin II type 1 receptors. Cell, 9(7), 1688. 10.3390/cells9071688 PMC7407103 32674346

[phy215382-bib-0011] Figueroa, C. D. , Dietze, G. , & Muller‐Esterl, W. (1996). Immunolocalization of bradykinin B2 receptors on skeletal muscle cells. Diabetes, 45, S24–S28. 10.2337/diab.45.1.s24 8529796

[phy215382-bib-0012] Folland, J. , Leach, B. , Little, T. , Hawker, K. , Myerson, S. , Montgomery, H. , & Jones, D. (2000). Angiotensin‐converting enzyme genotype affects the response of human skeletal muscle to functional overload. Experimental Physiology, 85(5), 575–579. 11038409

[phy215382-bib-0013] Fujimoto, N. , Prasad, A. , Hastings, J. L. , Arbab‐Zadeh, A. , Bhella, P. S. , Shibata, S. , Palmer, D. , & Levine, B. D. (2010). Cardiovascular effects of 1 year of progressive and vigorous exercise training in previously sedentary individuals older than 65 years of age. Circulation, 122(18), 1797–1805. 20956204 10.1161/CIRCULATIONAHA.110.973784 PMC3730488

[phy215382-bib-0014] Giaccaglia, V. , Nicklas, B. , Kritchevsky, S. , Mychalecky, J. , Messier, S. , Bleecker, E. , & Pahor, M. (2008). Interaction between angiotensin converting enzyme insertion/deletion genotype and exercise training on knee extensor strength in older individuals. International Journal of Sports Medicine, 29(1), 40–44. 17614015 10.1055/s-2007-964842

[phy215382-bib-0015] Gordon, S. E. , Davis, B. S. , & Carlson, C. J. (2001). Booth FW. ANG II is required for optimal overload‐induced skeletal muscle hypertrophy. American Journal of Physiology. Endocrinology and Metabolism, 280(1 43–1), 150–159. 10.1152/ajpendo.2001.280.1.E150 11120669

[phy215382-bib-0016] Habouzit, E. , Richard, H. , Sanchez, H. , Koulmann, N. , Serrurier, B. , Monnet, R. , Ventura‐Clapier, R. , & Bigard, X. (2009). Decreased muscle ACE activity enhances functional response to endurance training in rats, without change in muscle oxidative capacity or contractile phenotype. Journal of Applied Physiology, 107(1), 346–353. 19407247 10.1152/japplphysiol.91443.2008

[phy215382-bib-0017] Hutcheon, S. D. , Gillespie, N. D. , Crombie, I. K. , Struthers, A. D. , & McMurdo, M. E. T. (2002). Perindopril improves six minute walking distance in older patients with left ventricular systolic dysfunction: A randomised double blind placebo controlled trial. Heart, 88(4), 373–377. 12231595 10.1136/heart.88.4.373 PMC1767356

[phy215382-bib-0018] Izzo, J. L. , & Weir, M. R. (2011). Angiotensin‐converting enzyme inhibitors. Journal of Clinical Hypertension, 13(9), 667–675. 21896148 10.1111/j.1751-7176.2011.00508.x PMC8108813

[phy215382-bib-0019] Joyner, M. J. , & Coyle, E. F. (2008). Endurance exercise performance: The physiology of champions. The Journal of Physiology, 586(1), 35–44. 17901124 10.1113/jphysiol.2007.143834 PMC2375555

[phy215382-bib-0020] Kim, Y. C. , Mungunsukh, O. , McCart, E. A. , Roehrich, P. J. , Yee, D. K. , & Day, R. M. (2014). Mechanism of erythropoietin regulation by angiotensin II. Molecular Pharmacology, 85(6), 898–908. 24695083 10.1124/mol.113.091157

[phy215382-bib-0021] Kodama, S. , Saito, K. , Tanaka, S. , Maki, M. , Yachi, Y. , Asumi, M. , Sugawara, A. , Totsuka, K. , Shimano, H. , Ohashi, Y. , Yamada, N. , & Sone, H. (2009). Cardiorespiratory fitness as a quantitative predictor of all‐cause mortality and cardiovascular events in healthy men and women: A meta‐analysis. Journal of the American Medical Association., 301(19), 2024–2035. 19454641 10.1001/jama.2009.681

[phy215382-bib-0022] Kyhl, K. , Ahtarovski, K. A. , Iversen, K. , Thomsen, C. , Vejlstrup, N. , Engstrøm, T. , & Madsen, P. L. (2013). The decrease of cardiac chamber volumes and output during positive‐pressure ventilation. American Journal of Physiology. Heart and Circulatory Physiology, 305(7), 1004–1009. 10.1152/ajpheart.00309.2013 23893161

[phy215382-bib-0023] Leon, A. S. , Rice, T. , Mandel, S. , Despres, J. P. , Bergeron, J. , Gagnon, J. , Rao, D. C. , Skinner, J. S. , Wilmore, J. H. , & Bouchard, C. (2000). Blood lipid response to 20 weeks of supervised exercise in a large biracial population: The HERITAGE Family Study. Metabolism, 49(4), 513–520. 10778878 10.1016/s0026-0495(00)80018-9

[phy215382-bib-0024] Li, X. , Ooi, F. K. , Zilfalil, B. A. , & Yusoff, S. (2017). The influence of angiotensin‐converting enzyme gene ID polymorphism on human physical fitness performance in European and other populations. Sport Sciences for Health., 13(3), 495–506.

[phy215382-bib-0025] Lin, M. H. , Tseng, C. H. , Tseng, C. C. , Huang, C. H. , Chong, C. K. , & Tseng, C. P. (2001). Real‐time PCR for rapid genotyping of angiotensin‐converting enzyme insertion/deletion polymorphism. Clinical Biochemistry, 34(8), 661–666. 11849627 10.1016/s0009-9120(01)00281-8

[phy215382-bib-0026] Ma, F. , Yang, Y. , Li, X. , Zhou, F. , Gao, C. , Li, M. , & Gao, L. (2013). The association of sport performance with ACE and ACTN3 genetic polymorphisms: A systematic review and meta‐analysis. PLoS One, 8(1), 1–9. 10.1371/journal.pone.0054685 PMC3554644 23358679

[phy215382-bib-0027] MacInnis, M. J. , & Gibala, M. J. (2017). Physiological adaptations to interval training and the role of exercise intensity. The Journal of Physiology, 595(9), 2915–2930. 27748956 10.1113/JP273196 PMC5407969

[phy215382-bib-0028] Mahjoub, H. , Le Blanc, O. L. , Paquette, M. , Imhoff, S. , Labrecque, L. , Drapeau, A. , Poirier, P. , Bédard, É. , Pibarot, P. , & Brassard, P. (2019). Cardiac remodeling after six weeks of high‐intensity interval training to exhaustion in endurance‐trained men. American Journal of Physiology. Heart and Circulatory Physiology, 317(4), H685–H694. 31347913 10.1152/ajpheart.00196.2019

[phy215382-bib-0029] Mahmoudpour, S. H. , Asselbergs, F. W. , Souverein, P. C. , de Boer, A. , & Maitland‐van der Zee, A. H. (2018). Prescription patterns of angiotensin‐converting enzyme inhibitors for various indications: A UK population‐based study. British Journal of Clinical Pharmacology, 84(10), 2365–2372. 29943849 10.1111/bcp.13692 PMC6138483

[phy215382-bib-0030] Maron, B. J. , & Pelliccia, A. (2006). The heart of trained athletes: Cardiac remodeling and the risks of sports, including sudden death. Circulation, 114(15), 1633–1644. 17030703 10.1161/CIRCULATIONAHA.106.613562

[phy215382-bib-0031] Massidda, M. , Scorcu, M. , & Calò, C. M. (2014). New genetic model for predicting phenotype traits in sports. International Journal of Sports Physiology and Performance, 9(3), 554–560. 23982956 10.1123/ijspp.2012-0339

[phy215382-bib-0032] Matsumoto, T. , Ozono, R. , Oshima, T. , Matsuura, H. , Sueda, T. , Kajiyama, G. , & Kambe, M. (2000). Type 2 angiotensin II receptor is downregulated in cardiomyocytes of patients with heart failure. Cardiovascular Research, 46(1), 73–81. 10727655 10.1016/s0008-6363(00)00008-0

[phy215382-bib-0033] McBride, T. A. (2006). AT1 receptors are necessary for eccentric training‐induced hypertrophy and strength gains in rat skeletal muscle. Experimental Physiology, 91(2), 413–421. 16317083 10.1113/expphysiol.2005.032490

[phy215382-bib-0034] McGee, S. L. , & Hargreaves, M. (2020). Exercise adaptations: molecular mechanisms and potential targets for therapeutic benefit. Nature Reviews. Endocrinology, 16(9), 495–505. 10.1038/s41574-020-0377-1 32632275

[phy215382-bib-0035] Minami, N. , Li, Y. , Guo, Q. , Kawamura, T. , Mori, N. , Nagasaka, M. , Ogawa, M. , Ito, O. , Kurosawa, H. , Kanazawa, M. , & Kohzuki, M. (2007). Effects of angiotensin‐converting enzyme inhibitor and exercise training on exercise capacity and skeletal muscle. Journal of Hypertension, 25(6), 1241–1248. 17563537 10.1097/HJH.0b013e3280e126bf

[phy215382-bib-0036] Minshall, R. D. , Nakamura, F. , Becker, R. P. , & Rabito, S. F. (1995). Characterization of bradykinin B2 receptors in adult myocardium and neonatal rat cardiomyocytes. Circulation Research, 76(5), 773–780. 7728994 10.1161/01.res.76.5.773

[phy215382-bib-0037] Montero, D. , & Lundby, C. (2019). Regulation of red blood cell volume with exercise training. Comprehensive Physiology, 9(1), 149–164. 10.1002/cphy.c180004 30549016

[phy215382-bib-0038] Montgomery, H. E. , Marshall, R. , Hemingway, H. , Myerson, S. , Clarkson, P. , Dollery, C. , Hayward, M. , Holliman, D. E. , Jubb, M. , World, M. , Thomas, E. L. , Brynes, A. E. , Saeed, N. , Barnard, M. , Bell, J. D. , Prasad, K. , Rayson, M. , Talmud, P. J. , & Humphries, S. E. (1998). Human gene for physical performance. Nature, 393(6682), 221–222. 9607758 10.1038/30374

[phy215382-bib-0039] Perazella, M. , McPhedran, P. , Kliger, A. , Lorber, M. , Levy, E. , & Bia, M. J. (1995). Enalapril treatment of posttransplant erythrocytosis: efficacy independent of circulating erythropoietin levels. American Journal of Kidney Diseases, 26(3), 495–500. 7645558 10.1016/0272-6386(95)90496-4

[phy215382-bib-0040] Pickering, T. G. , Miller, N. H. , Ogedegbe, G. , Krakoff, L. R. , Artinian, N. T. , & Goff, D. (2008). Call to action on use and reimbursement for home blood pressure monitoring: Executive summary: A joint scientific statement from the american heart association, american society of hypertension, and preventive cardiovascular nurses association. Hypertension, 52(1), 1–9. 18497371 10.1161/HYPERTENSIONAHA.107.189011

[phy215382-bib-0041] Pratt, M. , Lewis‐Barned, N. , Walker, R. , Bailey, R. , Shand, B. , & Livesey, J. (1992). Effect of angiotensin converting enzyme inhibitors on erythropoietin concentrations in healthy volunteers. British Journal of Clinical Pharmacology, 34(4), 363–365. 1457271 10.1111/j.1365-2125.1992.tb05644.x PMC1381421

[phy215382-bib-0042] Puthucheary, Z. , Skipworth, J. R. A. , Rawal, J. , Loosemore, M. , Van Someren, K. , & Montgomery, H. E. (2011). The ACE gene and human performance: 12 years on. Sports Medicine, 41(6), 433–448. 21615186 10.2165/11588720-000000000-00000

[phy215382-bib-0043] Rigat, B. , Hubert, C. , Alhenc‐Gelas, F. , Cambien, F. , Corvol, P. , & Soubrier, F. (1990). An insertion/deletion polymorphism in the angiotensin I‐converting enzyme gene accounting for half the variance of serum enzyme levels. The Journal of Clinical Investigation, 86(4), 1343–1346. 1976655 10.1172/JCI114844 PMC296868

[phy215382-bib-0044] Roth, G. A. , Mensah, G. A. , Johnson, C. O. , Addolorato, G. , Ammirati, E. , Baddour, L. M. , Barengo, N. C. , Beaton, A. Z. , Benjamin, E. J. , Benziger, C. P. , Bonny, A. , Brauer, M. , Brodmann, M. , Cahill, T. J. , Carapetis, J. , Catapano, A. L. , Chugh, S. S. , Cooper, L. T. , Coresh, J. , … Fuster, V. (2020). Global burden of cardiovascular diseases and risk factors, 1990‐2019: Update from the GBD 2019 study. Journal of the American College of Cardiology, 76(25), 2982–3021. 33309175 10.1016/j.jacc.2020.11.010 PMC7755038

[phy215382-bib-0045] Saltin, B. , Nazar, K. , Costill, D. L. , Stein, E. , Jansson, E. , Essén, B. , & Gollnick, P. D. (1976). The nature of the training response; peripheral and central adaptations to one‐legged exercise. Acta Physiologica Scandinavica, 96(3), 289–305. 132082 10.1111/j.1748-1716.1976.tb10200.x

[phy215382-bib-0046] Scafoglieri, A. , & Clarys, J. P. (2018). Dual energy X‐ray absorptiometry: gold standard for muscle mass? Journal of Cachexia, Sarcopenia and Muscle, 9(4), 786–787. 29786955 10.1002/jcsm.12308 PMC6104103

[phy215382-bib-0047] Schmidt, W. , & Prommer, N. (2005). The optimised CO‐rebreathing method: A new tool to determine total haemoglobin mass routinely. European Journal of Applied Physiology, 95(5–6), 486–495. 16222540 10.1007/s00421-005-0050-3

[phy215382-bib-0048] Snell, P. G. , Stray‐Gundersen, J. , Levine, B. D. , Hawkins, M. N. , & Raven, P. B. (2007). Maximal oxygen uptake as a parametric measure of cardiorespiratory capacity. Medicine and Science in Sports and Exercise, 39(1), 103–107. 17218891 10.1249/01.mss.0000241641.75101.64

[phy215382-bib-0049] Spira, D. , Walston, J. , Buchmann, N. , Nikolov, J. , Demuth, I. , Steinhagen‐Thiessen, E. , Eckardt, R. , & Norman, K. (2016). Angiotensin‐converting enzyme inhibitors and parameters of sarcopenia: relation to muscle mass, strength and function: data from the Berlin Aging Study‐II (BASE‐II). Drugs & Aging, 33(11), 829–837. 27665105 10.1007/s40266-016-0396-8

[phy215382-bib-0050] Su, J. B. , Barbe, F. , Crozatier, B. , Campbell, D. J. , & Hittinger, L. (1999). Increased bradykinin levels accompany the hemodynamic response to acute inhibition of angiotensin‐converting enzyme in dogs with heart failure. Journal of Cardiovascular Pharmacology, 34(5), 700–710. 10547087 10.1097/00005344-199911000-00012

[phy215382-bib-0051] Sumukadas, D. , Band, M. , Miller, S. , Cvoro, V. , Witham, M. , Struthers, A. , McConnachie, A. , Lloyd, S. M. , & McMurdo, M. (2014). Do ACE inhibitors improve the response to exercise training in functionally impaired older adults? A randomized controlled trial. The Journals of Gerontology. Series A, Biological Sciences and Medical Sciences, 69(6), 736–743. 24201696 10.1093/gerona/glt142 PMC4022094

[phy215382-bib-0052] Sumukadas, D. , Witham, M. D. , Struthers, A. D. , & McMurdo, M. E. T. (2007). Effect of perindopril on physical function in elderly people with functional impairment: A randomized controlled trial. CMAJ, 177(8), 867–874. 17923654 10.1503/cmaj.061339 PMC1995143

[phy215382-bib-0053] Watanabe, T. , Owashi, K. , Kanauchi, Y. , Mura, N. , Takahara, M. , & Ogino, T. (2005). The short‐term reliability of grip strength measurement and the effects of posture and grip span. The Journal of Hand Surgery, 30(3), 603–609. 15925174 10.1016/j.jhsa.2004.12.007

[phy215382-bib-0054] Westerkamp, C. M. , Gordon, S. E. , & Gordon, S. E. (2005). Angiotensin‐converting enzyme inhibition attenuates myonuclear addition in overloaded slow‐twitch skeletal muscle. American Journal of Physiology. Regulatory, Integrative and Comparative Physiology, 289, 1223–1231. 10.1152/ajpregu.00730.2004 15961527

[phy215382-bib-0055] Williams, C. J. , Williams, M. G. , Eynon, N. , Ashton, K. J. , Little, J. P. , Wisloff, U. , & Coombes, J. S. (2017). Genes to predict VO2max trainability: A systematic review. BMC Genomics, 18(Suppl 8), 831. 10.1186/s12864-017-4192-6 29143670 PMC5688475

[phy215382-bib-0056] Yang, H. Y. T. , Erdös, E. G. , & Levin, Y. (1970). A dipeptidyl carboxypeptidase that converts angiotensin I and inactivates bradykinin. Biochimica et Biophysica Acta, 214(2), 374–376. 4322742 10.1016/0005-2795(70)90017-6

